# Take PART: development and usability evaluation of a computerized assessment of functional autonomy and participation in adolescents

**DOI:** 10.1186/s41687-026-01153-7

**Published:** 2026-07-20

**Authors:** Liron Lamash, Merav Porath, Efrat Landes, Yael Fogel

**Affiliations:** 1 Department of Occupational Therapy, University of Haifa, Haifa, Israel; 2 Ministry of Education, Haifa, Israel; 3 Department of Occupational Therapy, Ariel University, Ariel, Israel

**Keywords:** Adolescence, Functional autonomy, Participation, Assessment, Usability

## Abstract

**Background:**

Adolescence is a critical developmental period characterized byincreasing independence, functional autonomy, and participation across lifedomains. Despite the importance of supporting adolescents in reflecting ontheir functioning and identifying meaningful goals, few adolescent-centeredtools enable self-assessment of functional autonomy and participation inengaging, digitally accessible formats. This study aimed to develop Take PART(Participation and Activities Rating for Teenagers), a computerized assessmentof functional autonomy and participation, and evaluate its usability.

**Methods:**

The development of Take PARTwas conducted in two phases. Phase 1 focused on content generation and platformdevelopment using adolescent-reported activities, expert review, and auser-centered design process. Activities were organized into five life domains:self-care; daily activities at home and in the community; academicparticipation; work and volunteering; and leisure/social participation. Phase 2evaluated usability among 119 adolescents aged 12–18 years and 50 examinersusing the Post-Study System Usability Questionnaire (PSSUQ).

**Results:**

Adolescent reports informed the development of a 65-itemassessment reflecting everyday participation and functional autonomy. Expertreview contributed to the refinement of the domains, items, and platformstructure. The final assessment supports individualized domain selection andcollaborative goal-setting. Usability findings indicated high levels of systemusefulness, information quality, and interface quality among both adolescentsand examiners.

## Background

Adolescence represents a critical developmental period characterized by physical, cognitive, and emotional transformations as well as a gradual shift toward self-management and autonomy. It is a time when individuals begin to take greater responsibility for their daily lives, establish personal goals, and develop strategies for managing tasks, relationships, and transitions [1–3]. According to the World Health Organization [4], *adolescence* is the period between ages 10 and 19 years, marking the transition from childhood to adulthood. This period includes processes essential for preparing adolescents for adult roles and responsibilities, including independent living, vocational engagement, and participation in community life [5].

One primary developmental task of adolescence is the gradual transition from dependence on caregivers toward increased self-management, decision-making, and responsibility for everyday life activities [6, 7]. During this period, adolescents are expected to carry out daily tasks, solve problems, and make choices in adaptive and contextually appropriate ways. However, this transition often involves challenges related to executive functioning, emotional regulation, or navigating environmental demands, which may complicate the development of autonomous functioning [8, 9]. These processes occur within dynamic contexts of home, school, and community, where environmental supports and constraints shape adolescents’ opportunities for engagement and growth [10]. Accordingly, adolescence represents a critical window for fostering skills related to autonomy and participation, as well as for supporting adolescents in reflecting on their abilities and setting personally meaningful goals [7, 11]. While *independence* is commonly defined as the ability to perform activities without assistance, *functional autonomy* is a broader, more complex construct. Beyond task execution, functional autonomy encompasses adolescents’ capacity for choice, initiative, self-regulation, and responsibility in managing daily life activities [8, 12]. It reflects the ability to decide whether, when, and how to engage in activities in ways that align with one’s goals and values, and involves both performance and motivation to assume responsibility for everyday functioning [13]. During adolescence, the development of functional autonomy includes negotiating increasing independence from caregivers while learning to manage complex routines and social expectations across multiple life domains [14, 15]. Importantly, autonomy is not a uniform or global construct; adolescents may differ in both their actual level of independence and their desire to manage specific tasks, and these variations often depend on the life domain involved, such as self-care, academic activities, household responsibilities, or leisure [16]. Despite its central role in adolescent development, functional autonomy is often underassessed, particularly from the adolescent’s own perspective. Many existing assessments rely on parental or teacher reports or focus on general adaptive functioning, without adequately capturing adolescents’ subjective experience of autonomy or their motivation for change [8, 17]. Yet, research suggests that involving adolescents in reflecting on their functioning and participating in goal-setting processes is associated with greater engagement, self-efficacy, and long-term independence [18, 19]. Because functional autonomy is expressed through adolescents’ engagement in everyday activities and roles, it is closely linked to participation in daily life contexts.

*Participation*, defined as involvement in life situations, is widely recognized as a central dimension of health, well-being, and functional development during adolescence [20]. However, participation does not necessarily imply active engagement or personal agency, as adolescents may be present in activities due to external arrangements without exercising choice or initiative. Consistent with the ICF-CY framework, functional autonomy reflects the extent to which participation is characterized by choice, initiative, self-regulation, and responsibility, shaping whether involvement is experienced as passive presence or as intentional, self-directed engagement. As adolescents develop greater capacity to manage their daily routines, functional autonomy may support their ability to navigate various life situations and roles, which are essential to personal growth and social inclusion [20, 21].

Despite growing recognition of the importance of functional autonomy and participation during adolescence, existing assessment tools tend to address these constructs only partially. Widely used instruments developed within adaptive behavior frameworks, such as the Adaptive Behavior Assessment System, the Vineland Adaptive Behavior Scales, and the Pediatric Evaluation of Disability Inventory, provide valuable information about observable skills and functional competence, primarily based on proxy reports [22–24]. While adaptive behavior and functional autonomy are related constructs, they are not synonymous. Adaptive behavior assessments focus on whether adolescents are able to perform daily tasks and meet environmental expectations, whereas functional autonomy emphasizes how adolescents experience and manage their functioning, including dimensions of choice, initiative, self-regulation, and responsibility in everyday life. Consequently, these instruments place limited emphasis on adolescents’ subjective experience of autonomy and their active role in directing participation across daily contexts. Several self-report measures have advanced the field by incorporating adolescents’ perspectives and emphasizing participation within everyday routines and contexts, including the DAY-Opp, the DRA, and the PEM-CY [8, 20, 25]. These tools offer important insights into activity performance, independence, satisfaction, and environmental influences. However, they typically focus on describing what adolescents do and how often, rather than how participation is experienced, how activities reflect personal priorities and agency, or how adolescents identify and prioritize areas for change. In addition, these measures generally apply uniform item sets across broad age ranges, with limited sensitivity to developmental stage or gender-related differences in roles, expectations, and daily experiences. Together, these limitations suggest that existing measures capture important components of adolescent functioning and participation but provide an incomplete picture of functional autonomy in everyday life.

Specifically, there remains a need for assessment approaches that not only capture adolescents’ functioning across everyday life domains, but also incorporate their subjective perspectives regarding autonomy, priorities, and desired areas for change. Such approaches should be sensitive to developmental and gender-related variability, support adolescent involvement in identifying meaningful goals, and remain accessible and engaging for individuals with diverse cognitive and communicative profiles.

Beyond content-related limitations, existing assessment tools are often not fully aligned with adolescents’ developmental, cognitive, and technological realities. Many widely used measures rely on text-based, static questionnaires, which may limit adolescents’ engagement and fail to capture the diversity and dynamic nature of their everyday experiences [24, 26]. Growing evidence indicates that digital, interactive, and visually enriched formats enhance adolescents’ attention, motivation, and involvement in assessment and intervention contexts [27]. Tools that incorporate images, icons, or dynamic interfaces are particularly suited to adolescents, who are accustomed to visual communication and technology-driven environments [28]. Such formats support identification with real-life scenarios, reduce cognitive load, and promote more nuanced reflection on personal experiences [29]. In addition, modular assessment structures are increasingly recognized as essential during adolescence, given the wide variability in roles, expectations, and readiness to address different life domains [7]. Modular designs allow adolescents to exercise choice and prioritize meaningful areas, thereby supporting autonomy and relevance in the assessment process [11, 21]. Accessibility represents a further critical consideration. Adolescents with neurodevelopmental conditions, learning difficulties, or executive functioning challenges may experience particular barriers when completing traditional, text-heavy assessments. Visual and interactive tools provide more intuitive, low-threshold ways to express preferences, abilities, and goals, thereby broadening participation and inclusivity in assessment processes [8, 30].

In summary, there is a pressing need for adolescent-centered assessment tools that capture functional autonomy as it is enacted within everyday participation contexts. Such tools should integrate adolescents’ subjective perspectives, support choice and prioritization, be sensitive to developmental and gender-related variability, and be delivered in formats that are engaging, accessible, and aligned with adolescents’ cognitive and technological realities. Such information is important for identifying meaningful intervention targets, supporting collaborative goal-setting, and monitoring adolescents’ development of autonomy and participation across everyday contexts.

The Take PART (Participation and Activities Rating for Teenagers) was developed to address these gaps and is conceptually grounded in the International Classification of Functioning, Disability and Health for Children and Youth (ICF-CY). Within this framework, functional autonomy is understood as a personal factor that shapes how adolescents engage in activities and participate in life situations through choice, initiative, self-regulation, and responsibility. Accordingly, Take PART assesses functional autonomy as it is enacted within core activity and participation domains relevant to adolescence, including self-care, instrumental daily activities, academic engagement, work and volunteering, and leisure and social participation. Take PART is a computerized, self-report assessment that incorporates real-life activity contexts, visual representations, and interactive rating formats to support adolescents’ reflection on their current performance and priorities for change. By directly involving adolescents in evaluating their own functioning and priorities, the assessment is intended to support adolescent engagement in the assessment process, facilitate collaborative goal-setting, and help identify meaningful targets for intervention. The tool is designed to be age- and gender-sensitive, acknowledging the heterogeneity of adolescents’ roles, expectations, and developmental trajectories. Although developed within the Israeli cultural and educational context, Take PART was designed to reflect broad domains of adolescent functioning and participation, supporting its potential applicability and adaptation across cultural settings. The assessment was designed to be broadly applicable across adolescent populations and may be particularly relevant for future use with adolescents with neurodevelopmental conditions, given its visual, interactive, and flexible structure.

The present study aimed to describe the development of Take PART and evaluate its usability among adolescents and examiners. Specifically, the study aimed to:

1. Describe the development process of Take PART, including content generation, domain and item development, expert review, and platform design.
2. Explore the usability and accessibility of Take PART as perceived by adolescents and examiners using the Post-Study System Usability Questionnaire (PSSUQ).

## Method

### Study design

This study was conducted in two phases. Phase 1 focused on developing the Take PART assessment through content generation, expert review, and platform development. Phase 2 involved a cross-sectional evaluation of the assessment’s usability and accessibility among typically developing adolescents and examiners. All data were collected at a single time point using an online platform. Adolescents completed the Take PART assessment, demographic questions, and the Post-Study System Usability Questionnaire (PSSUQ), while examiners completed the PSSUQ following their experience administering the assessment and using the platform.

### Phase 1: Development of Take PART

Phase 1 focused on developing the Take PART assessment through content generation, expert review, and platform development, whereas Phase 2 focused on evaluating the usability and accessibility of the completed assessment.

*Content Development Process and Evidence Supporting Content Validity.* Existing instruments primarily rely on parental or teacher reports, often failing to capture adolescents’ own perceptions of their abilities and participation in daily life [20–24]. Thus, Take PART was developed to address a significant gap in adolescent assessment tools by providing a self-report measure of functional autonomy. The development followed a multi-phase, user-centered approach that integrated input from adolescents and professionals who work with adolescents to ensure the assessment would be relevant to adolescents’ everyday experiences and developmental needs.

Ecological validity was a guiding principle throughout the development process. Item content was derived from adolescents’ self-reported everyday activities across multiple life domains, collected through an initial survey of adolescents aged 12–18 years. The content development process was guided by the International Classification of Functioning, Disability and Health for Children and Youth (ICF-CY) framework [4]. The development process was designed to generate evidence supporting the assessment’s content validity through iterative item generation, expert review, and refinement with adolescents and occupational therapy experts. The outcomes of this development process are presented in the Results section.

*Digital Platform and Assessment Flow.* The Take PART was developed as a customized web-based application, designed to provide a flexible, accessible, and user-friendly platform for evaluating adolescents’ functional autonomy. The system was built with NodeJS and ReactJS and deployed on Netlify (https://www.netlify.com), enabling secure remote access from various devices, including computers, tablets, and smartphones. The development process included creating three distinct environments, each designed to serve specific user roles:

1. The *user environment* provides adolescents with a structured assessment flow. At the beginning of the assessment, adolescents complete a series of introductory screens in which they enter their name, age, and gender. They are then asked to select the life domains to be evaluated. The Take PART assessment allows flexible domain selection, either collaboratively by the adolescent and clinician or based on clinical judgment, enabling focused assessment of relevant areas while avoiding evaluation of domains in which the adolescent is already functioning well. Following domain selection, adolescents are presented with a slide-based interface to rate their performance and importance of change across activities, using a 5-point scale accompanied by visual representations and verbal descriptions of success and difficulty in daily tasks. For research purposes in the current study, all adolescents completed all domains.
2. The *examiner environment* allows professionals to view assessment results and generate automated reports for individual adolescents or groups of participants. As Take PART is a self-report assessment, the examiner’s role is limited to supporting administration when needed and reviewing the results together with the adolescent to facilitate shared interpretation and identification of intervention priorities. The examiner’s back-end environment primarily serves as a centralized workspace that aggregates assessment data for all adolescents registered under a given clinician.
3. The *administrator environment* features a management dashboard that enables secure data storage, categorized viewing, and export of assessment results for clinical and research purposes. In addition, this environment allows system administrators and developers to edit textual content and visual materials, including activity descriptions and images, and to adjust the structure and organization of life domains. This administrative flexibility supports ongoing maintenance, updates, and contextual adaptation of the Take PART assessment while preserving data security and system integrity.

*Interface Design and Accessibility.* As part of the system design, representative images were incorporated to enhance user engagement and support accessibility for adolescents with diverse cognitive and communication abilities. Each activity is illustrated by paired images depicting successful and difficult task performance, facilitating comprehension across a broad range of users. The images were generated using AI-based graphic tools, including paid applications, to ensure age-, gender-, and culturally appropriate representations and to promote ethnic and social diversity.

The platform’s visual design was developed in consultation with adolescents and examiners to ensure that the interface is intuitive, accessible, and engaging. Based on stakeholder feedback, a clean and largely monochromatic color scheme was adopted to minimize visual overload and enhance clarity.

**Fig. 1:**
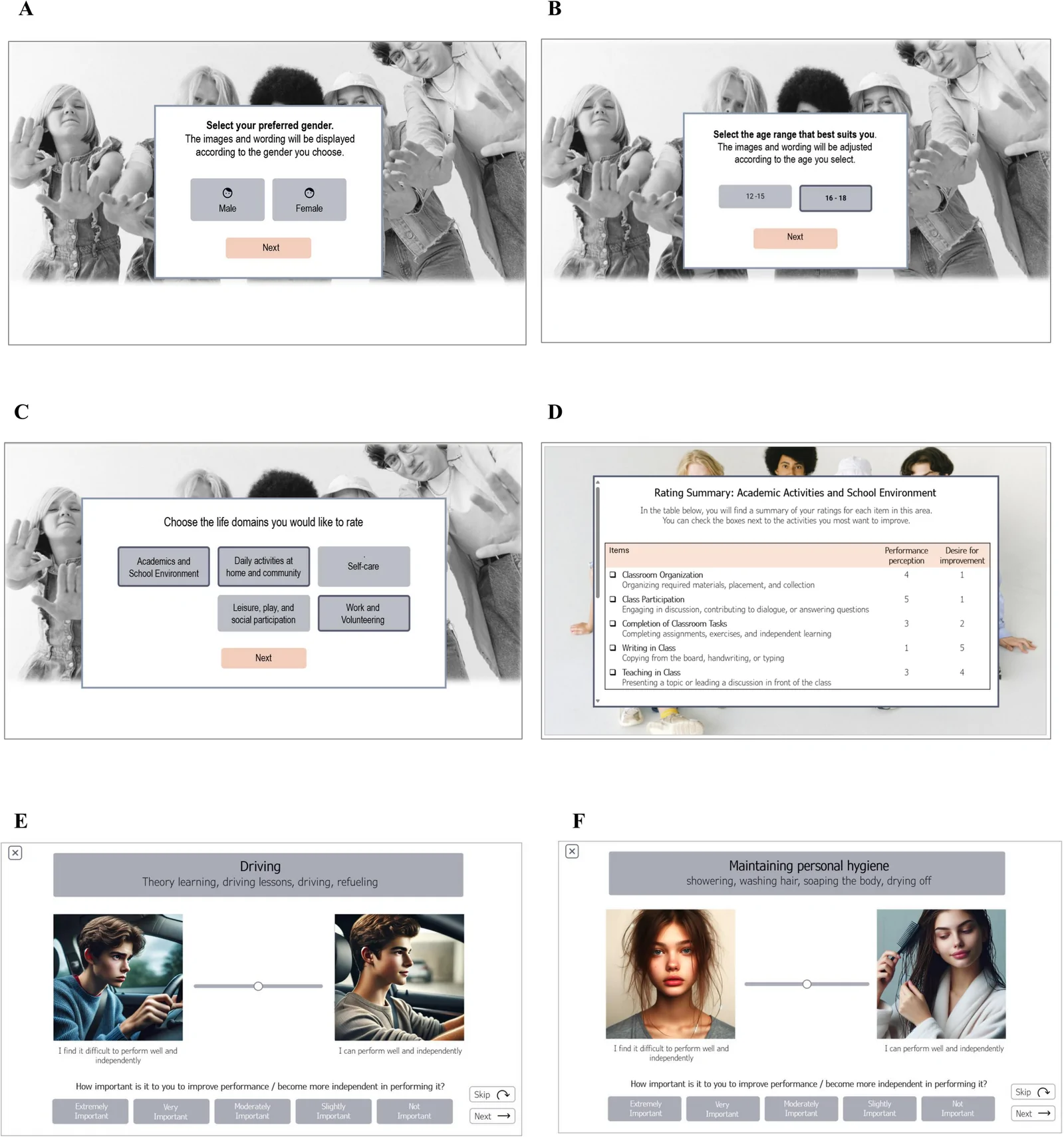
Screenshots of the Take PART adolescent interface. *Note*. **A** = gender selection, **B** = age selection, **C** = domain selection, **D** = domain rating summary, **E** = sample item-daily activities (older boys), **F** = sample item-self-care (all ages girls)

For each item, adolescents rate their performance using an interactive slider presented as a continuous line without numeric markers. The slider is anchored by two images depicting unsuccessful performance on one end and successful performance on the other, allowing adolescents to position the marker according to their perceived level of functioning. Slider positions are automatically converted into numerical scores ranging from 1 (significant difficulty or dependence) to 5 (fully independent and efficient performance). In addition, adolescents rate the importance of improving their performance or autonomy for each activity using a 5-point Likert scale ranging from 1 (not important at all) to 5 (very important). Items that are not relevant to the adolescent’s daily routine may be skipped. Upon completion of each domain, a summary screen displays performance and importance ratings, after which adolescents are asked to select two to three priority activities for intervention planning. Screenshots of the Take PART adolescent interface are presented in Fig. 1.

The Take PART platform includes distinct interfaces for adolescents, examiners, and administrators, supporting self-report assessment, result review, and system management. This structure allows flexible clinical use while maintaining scalability and alignment with clinical and educational contexts.

### Phase 2: Usability evaluation

#### Participants

A total of 119 adolescents aged 12 to 18 years (*M* = 15.16, SD = 2.05), attending middle and high school in grades 7–12, participated in the study, which was conducted in Israel. Participants were recruited from different regions across the country. Of these, 46 identified as male (38.7%), 72 as female (60.5%), and one participant as non-binary (0.8%); 104 (87.4%) were enrolled in regular mainstream classes, eligible for a full matriculation diploma. Nine participants (7.6%) reported attending either vocational or gifted education classes, and six participants (5%) did not provide information on this item. Fifty-eight participants (48.7%) reported living in urban areas, and 61 (51.3%) resided in towns or rural settlements.

The usability evaluation also included 50 final-year occupational therapy students who served as examiners. All had completed most of their required clinical training hours. Data collection occurred as part of an academic course focused on adolescent functioning and learning. All examiners received structured training in administering the Take PART assessment and using the platform to ensure consistent implementation across participants.

#### Tools

##### Demographic information

The adolescents answered demographic questions, including age, sex (male/female), place of residence, and educational setting.

##### Post-study system usability Questionnaire

We used the PSSUQ short version [31] to assess the perceived usability of the Take PART computerized system among adolescents and examiners, with a focus on its technological and interface-related aspects. The short version consists of 16 items rated on a 7-point Likert scale from 1 (strongly agree) to 7 (strongly disagree). The PSSUQ provides a total usability score calculated by averaging all items as well as scores for three subscales: system usefulness, information quality, and interface quality. In this scoring system, lower scores reflect higher perceived usability. According to benchmark data reported by Sauro and Lewis [32], PSSUQ scores across usability studies range from 2.49 to 3.02 across subscales, with an overall mean of 2.82. Lower scores indicate more favorable usability ratings. The PSSUQ has demonstrated strong psychometric properties, including excellent internal consistency for the total scale and subscales (Cronbach’s α = 0.97 overall and 0.91–0.96 for subscales) and evidence of criterion validity [31, 32].

### Procedure

This study was approved by the Ethics Committee of [affiliations blinded for peer review] and the Ethics Committee of [affiliations blinded for peer review]. Examiners recruited participants using convenience and snowball sampling methods via personal networks and social media, contacting the parents of adolescents who met the study’s inclusion criteria. To ensure that the sample consisted solely of typically developing adolescents, children whose parents reported any neurodevelopmental or health diagnoses (e.g., autism spectrum disorder, cognitive impairment, mental health conditions, or significant motor disabilities) were excluded from the study. Parents provided written informed consent for their child’s participation and agreed to share their child’s contact details with the examiners. Each adolescent was then contacted directly, received an explanation of the study procedures, and provided informed assent prior to participation.

All adolescents attended a single-session assessment conducted in a quiet, distraction-free environment, lasting approximately 1 hour. Adolescents independently completed the Take PART assessment using the computerized platform. Examiners provided standardized instructions using a unified administration script to ensure consistency across participants. Upon completion of the assessment, adolescents completed the Post-Study System Usability Questionnaire (PSSUQ) via Qualtrics, which required approximately 5 minutes.

As part of the usability evaluation, examiners underwent hands-on training and system exploration, during which they independently navigated the Take PART platform, administered the assessment to at least two adolescents, generated reports, and practiced data-export procedures. Following their experience with the platform, examiners completed the PSSUQ anonymously via Qualtrics, which required approximately 5 minutes.

### Data analysis

All statistical analyses were conducted using IBM SPSS Statistics version 28. Descriptive statistics were calculated to characterize participant demographics and summarize usability findings. Separate means and standard deviations were calculated for adolescents and examiners for the total PSSUQ score and its three subscales: system usefulness, information quality, and interface quality. Lower scores indicate higher perceived usability.

## Results

### Phase 1: Development outcomes

#### Adolescent-informed content generation

In the initial development phase, an anonymous survey was distributed to adolescents aged 12–18 years to identify activities and participation experiences that were meaningful and relevant to their everyday lives. Adolescents reported a wide range of everyday activities spanning self-care, household and community responsibilities, school-related tasks, leisure pursuits, social interactions, and early vocational experiences. Analysis of these responses also revealed developmental and gender-related differences. For example, older adolescents (16–18 years) more frequently reported activities related to driving and independent transportation, whereas adolescent girls more frequently reported activities related to personal grooming, hygiene, and menstrual care. These findings informed the initial activity pool and guided subsequent domain development and item tailoring.

#### Domain generation and expert review

The activities reported by adolescents were reviewed and organized through an iterative process involving the research team and an expert panel consisting of six occupational therapists with expertise in pediatric neurodevelopmental conditions, adolescent intervention, and clinical assessment. Guided by the International Classification of Functioning, Disability and Health for Children and Youth (ICF-CY) framework, activities were grouped into conceptually coherent domains representing major contexts of adolescent functioning and participation. This process resulted in five domains that captured both personal and contextual aspects of adolescents’ daily lives across home, school, community, vocational, and social settings.

The expert panel evaluated the relevance, clarity, developmental appropriateness, and clinical utility of the proposed activities and domains. Based on their feedback, activities were refined, reorganized, merged when overlap was identified, and modified to improve developmental relevance and clinical applicability. Age- and gender-specific adaptations were also incorporated when supported by adolescent reports and expert consensus. This process resulted in the final domain structure presented in Table 1.

**Table 1:** Final domain structure of the Take PART assessment

Domain	Domain Description	Examples of Activities Included	No. of Items
Self-care	Activities related to managing personal needs, health, and daily self-maintenance.	Personal hygiene, grooming, dressing, medication management, health-related routines.	11
Daily activities at home and in the community	Activities that support independent functioning within the home and community environments.	Household chores, shopping, money management, community mobility, independent transportation.	16
Academic and school participation	Activities associated with managing educational responsibilities and participating in school life.	Studying, homework completion, classroom participation, organization of school materials, time management.	14
Work and volunteering	Activities related to employment, volunteering, and preparation for future vocational roles.	Part-time work, volunteering, workplace responsibilities, job-related tasks.	7
Leisure and social participation	Activities that promote recreation, social engagement, and participation in meaningful free-time pursuits.	Hobbies, spending time with friends, recreational activities, online social activities, and community participation.	17

#### Final structure of Take PART

The final version of Take PART was designed as a modular computerized assessment that allows for flexible administration to meet adolescents’ needs and priorities. For each activity, adolescents rate their perceived level of performance and autonomy, as well as the importance of improving or changing their functioning in that area. Following completion of each domain, adolescents identify priority activities for future intervention and goal-setting. This structure was designed to capture not only adolescents’ current functioning, but also their perspectives regarding choice, priorities, and desired areas for change, reflecting key dimensions of functional autonomy.

### Phase 2: Usability of the Take PART

Overall, adolescents and examiners reported positive usability experiences with the Take PART assessment. The mean total PSSUQ score was 2.45 (SD = 1.04) among adolescents and 2.60 (SD = 1.44) among examiners. Both values were lower than the published normative PSSUQ benchmark score of 2.82 [32], indicating favorable perceptions of the system’s usability. Detailed usability results for adolescents and examiners are presented in Table 2.

**Table 2:** Adolescents and examiners’ usability ratings of the Take PART

Usability Aspect	Adolescents		Examiners	
	Range	*M* (*SD*)	Range	*M* (*SD*)
System usefulness	1.00–6.33	2.46 (1.19)	1.00–7.00	2.32 (1.57)
Information quality	1.00–5.67	2.32 (1.23)	1.00–7.00	2.78 (1.61)
Interface quality	1.00–7.00	2.64 (1.48)	1.00–7.00	2.74 (1.60)
**Total usability**	1.00–5.00	2.45 (1.04)	1.00–6.93	2.60 (1.44)

## Discussion

This study described the development and usability evaluation of Take PART, a novel digital self-report assessment designed to evaluate functional autonomy as it is enacted within adolescents’ everyday participation contexts. Developed to address limitations in existing adolescent assessments, Take PART integrates adolescents’ subjective perspectives with ecologically grounded activity domains and distinguishes between perceived performance, autonomy, and the personal importance of change across daily life areas. The findings highlight the value of an adolescent-informed, theory-driven development process and demonstrate favorable usability ratings among both adolescents and examiners. Together, these findings support the feasibility and potential clinical utility of Take PART as an adolescent-centered assessment tool.

### Development of Take PART

The Take PART tool was developed to address a well-recognized need for adolescent-centered, visually engaging, and ecologically valid assessments that capture real-life participation and functional autonomy. Traditional tools often rely on proxy reports from parents or teachers or present text-heavy questionnaires that may not align with adolescents’ cognitive preferences or lived experiences [20, 24]. In contrast, Take PART enables adolescents to self-report their current performance and their motivation for change across five key life domains: self-care, instrumental daily activities, academic engagement, work and volunteering, and play, leisure, and social participation. These domains align with the International Classification of Functioning, Disability and Health for Children and Youth [4] and the Occupational Therapy Practice Framework [10], emphasizing the interaction between individual abilities, daily life demands, and environmental contexts. A key strength of the development process was the direct involvement of adolescents in generating the activity content that formed the basis of the assessment. The resulting domains and activities reflected adolescents’ everyday experiences across home, school, community, vocational, and social contexts, while age- and gender-specific adaptations enhanced developmental relevance. This approach is consistent with contemporary developmental perspectives that emphasize adolescence as a critical period for increasing agency, self-management, and preparation for adult roles and responsibilities [33].

The tool’s development was grounded in user-centered design, integrating visual supports, intuitive sliders, and modular selection of items by age and gender to ensure developmental relevance and accessibility. This approach reflects current trends that advocate for interactive, digital, and image-based assessments to enhance engagement and reduce cognitive load, particularly for adolescents with diverse abilities [28, 29].

Crucially, Take PART bridges assessment and intervention by allowing adolescents not only to evaluate their performance but also to prioritize areas for improvement. This structure promotes self-determination and collaborative goal-setting in line with contemporary developmental frameworks [11, 15, 34]. Taken together, the theory-driven domain selection, adolescent-generated activity content, and expert-informed refinement process provide evidence supporting the assessment’s content validity while also enhancing the relevance and developmental appropriateness of its content and structure.

### Usability of the Take PART

The second aim of this study was to evaluate the usability of the Take PART assessment among adolescents and examiners. Overall, both groups reported favorable usability ratings, with mean total PSSUQ scores of 2.45 among adolescents and 2.60 among examiners. These values were lower than the published normative benchmark score of 2.82 reported by Sauro and Lewis [32], suggesting that Take PART provides a positive user experience for both end-users and professionals.

The favorable usability ratings are particularly important given the developmental characteristics of adolescence and the increasing use of digital technologies in assessment and intervention processes. Take PART was intentionally designed as a visual, interactive, and modular assessment that incorporates image-based representations, interactive rating formats, and flexible administration options. Such features are consistent with recommendations emphasizing the importance of accessible, engaging, and user-centered digital tools for adolescents, particularly when supporting reflection on everyday functioning and participation [28, 29, 35].

Importantly, usability was rated positively not only by adolescents, who completed the assessment, but also by examiners who administered the tool and reviewed the resulting information. This finding supports the feasibility of implementing Take PART within clinical and educational settings, where assessment tools must be acceptable to both adolescents and professionals in order to be successfully integrated into practice.

Taken together, the usability findings suggest that Take PART may offer a practical and engaging approach to assessing functional autonomy and participation. By combining adolescent self-report with an accessible digital format, the tool has the potential to support collaborative assessment, goal-setting, and intervention planning in a manner that is meaningful to adolescents and feasible for professionals.

### Conclusion and clinical implications

This study describes the development and usability evaluation of Take PART, a novel digital assessment designed to examine functional autonomy as it is enacted within adolescents’ everyday participation contexts. By embedding the assessment within real-world domains and incorporating age- and gender-sensitive content, Take PART addresses an important need for adolescent-centered tools that are ecologically valid, developmentally appropriate, and accessible.

Unlike many existing assessments, Take PART combines adolescents’ ratings of performance and autonomy with their perceptions of the importance of change, providing a more nuanced and adolescent-driven understanding of everyday functioning and participation. This approach allows adolescents to identify personally meaningful priorities and actively participate in the assessment process.

From a clinical perspective, Take PART has the potential to support collaborative goal-setting and shared decision-making by helping adolescents and clinicians identify areas of strength, challenges, and intervention priorities. The assessment’s modular structure and digital format may further facilitate its integration into clinical, educational, and community-based services. Taken together, the findings suggest that Take PART represents a promising approach for supporting adolescent-centered assessment and intervention planning.

### Limitations and future directions

Several limitations should be considered when interpreting the findings of this study. First, the current study focused on the development and usability evaluation of Take PART rather than on a comprehensive examination of its measurement properties. Content development was guided by a theory-driven, user-centered process that incorporated adolescent input and expert review. Although this approach is consistent with recommended practices for early-stage instrument development, future studies should further examine the assessment’s measurement properties using larger, more diverse samples.

Second, although the domain structure of Take PART was conceptually grounded in the ICF-CY framework and informed by adolescents’ reported experiences, the assessment’s underlying dimensional structure was not examined in the current study. Future research should evaluate the assessment across larger, more diverse adolescent populations and examine its use in various clinical, educational, and community settings.

Third, although the instrument is grounded in the ICF-CY framework, some activities and routines included in the current version reflect norms of the Israeli cultural and educational context in which the tool was developed. Therefore, caution is warranted when applying Take PART in other cultural settings without prior adaptation. Future studies should examine its cross-cultural applicability and conduct systematic cultural adaptation procedures.

The study also employed a cross-sectional design, which did not allow examination of responsiveness to change over time. Future research should evaluate the utility of Take PART for monitoring developmental progress, intervention outcomes, and changes in functional autonomy across adolescence.

In addition, the sample consisted solely of typically developing adolescents. Further research is needed to examine the applicability and usability of Take PART among adolescents with neurodevelopmental conditions, chronic health conditions, and other populations who may particularly benefit from adolescent-centered assessment approaches.

Although usability ratings were favorable, the study relied exclusively on quantitative usability measures. Incorporating qualitative methods, such as interviews, focus groups, or open-ended feedback, could provide richer insights into adolescents’ and clinicians’ experiences with the assessment and inform future refinements.

Future research should also explore the implementation of Take PART within clinical, educational, and community-based services and examine its contribution to assessment, collaborative goal-setting, intervention planning, and participation-related outcomes.

## Data Availability

The datasets collected and analyzed during the current study are not publicly available due to ethical restrictions, but are available from the corresponding author on reasonable request.

## Abbreviations

Take PART: Participation and Activities Rating for Teenagers
PSSUQ: Post-Study System Usability Questionnaire
